# When and How to Use Subcutaneous Antibiotics

**DOI:** 10.1093/cid/ciaf691

**Published:** 2026-01-20

**Authors:** Stefano Di Bella, Nicholas Geremia, Federico Pea, Markus Zeitlinger, Gianfranco Sanson, Jacopo Monticelli, Felix Bergmann, Christian Motet, Christophe Lambotte-Buffet, Verena Zerbato, Milo Gatti

**Affiliations:** Infectious Diseases Unit, Trieste University Hospital (ASUGI), Trieste, Italy; Clinical Department of Medical, Surgical and Health Sciences, Trieste University, Trieste, Italy; Department of Clinical Medicine, Infectious Diseases Unit, Ospedale “dell'Angelo”, Venice, Italy; Department of Clinical Medicine, Infectious Diseases Unit, Ospedale Civile “S.S. Giovanni e Paolo”, Venice, Italy; Department of Medical and Surgical Sciences, Alma Mater Studiorum University of Bologna, Bologna, Italy; Department for Integrated Infectious Risk Management, Clinical Pharmacology Unit, IRCCS Azienda Ospedaliero-Universitaria di Bologna, Bologna, Italy; Department of Clinical Pharmacology, Medical University of Vienna, Vienna, Austria; Department of Medical, Surgical and Health Science, University of Trieste, Trieste, Italy; Infectious Diseases Unit, Trieste University Hospital (ASUGI), Trieste, Italy; Department of Clinical Pharmacology, Medical University of Vienna, Vienna, Austria; Clinic of Infectious Diseases, Hôpital Universitaire de Bruxelles, Université Libre de Bruxelles, Brussels, Belgium; Department of Geriatrics, Hôpital Universitaire de Bruxelles, Université Libre de Bruxelles, Brussels, Belgium; Infectious Diseases Unit, Trieste University Hospital (ASUGI), Trieste, Italy; Department of Medical and Surgical Sciences, Alma Mater Studiorum University of Bologna, Bologna, Italy; Department for Integrated Infectious Risk Management, Clinical Pharmacology Unit, IRCCS Azienda Ospedaliero-Universitaria di Bologna, Bologna, Italy

**Keywords:** subcutaneous antibiotic administration, outpatient parenteral antimicrobial therapy (OPAT), pharmacokinetics/pharmacodynamics (PK/PD), intravenous-to-subcutaneous switch; antimicrobial delivery systems

## Abstract

Subcutaneous antibiotic administration is increasingly recognized as a valuable alternative to intravenous therapy in selected clinical contexts. It is particularly advantageous for patients with poor venous access, frail or cachectic individuals, and in outpatient or palliative care settings, when oral options are not feasible. Subcutaneous delivery of antibiotics with predominantly time-dependent activity, particularly β-lactams (eg ceftriaxone, ertapenem) and glycopeptides (teicoplanin), allows attainment of therapeutic pharmacokinetic/pharmacodynamic (PK/PD) targets comparable to intravenous administration, while maintaining stable concentrations and reducing catheter-related complications. By contrast, PK/PD benefits are limited for agents with more concentration-dependent activity, such as aminoglycosides and fluoroquinolones due to reduced peak levels and local toxicity, whereas daptomycin shows favorable exposure and target attainment with acceptable tolerability. Available evidence suggests good tolerability, although regulatory frameworks remain limited. This multidisciplinary review, authored by infectious disease specialists, clinical pharmacologists, and nurses, summarizes current clinical experience, PK/PD data, and technical aspects of subcutaneous infusion.

## Teaser

Subcutaneous administration of selected antibiotics provides a practical, safe alternative to intravenous therapy in patients with poor venous access, in outpatient or palliative care. This multidisciplinary review summarizes pharmacological data, clinical evidence, and technical considerations to guide clinical practice.

Key Points

- Subcutaneous route is valuable when intravenous access is limited.
- β-lactams, teicoplanin, and daptomycin show favorable profiles.
- Fluoroquinolones and aminoglycosides appear unsuitable.
- Subcutaneous antibiotics are useful in OPAT, frail or palliative patients.
- Practical considerations for safe clinical use provided.

Subcutaneous (SC) antibiotic administration is increasingly recognized as a valuable tool in infectious diseases, particularly when intravenous (IV) access is not fceasible or desirable [1, 2]. In daily practice, clinicians often manage patients with exhausted or thrombosed veins, central lines already used for other therapies, or with at-risk behaviors such as IV drug use or factitious disorders. SC delivery may offer a practical and safe alternative, including in outpatient setting, where avoiding central access, especially in frail, cachectic, or palliative patients, may reduce complications and improve quality of life, or when oral options are not feasible. Pharmacokinetic/pharmacodynamic (PK/PD) considerations support this approach: for time-dependent antibiotics, especially beta-lactams, SC administration can enhance the time free concentrations remain above the minimum inhibitory concentration (MIC) (%fT _>_  _MIC_), with more stable plasma concentrations and reduced fluctuation between peak (C_max_) and trough (C_min_) [3–5]. This favors not only efficacy but also tolerability. While C_max_ is reduced compared with IV, this is of little relevance in nonconcentration-dependent drugs. Additionally, for agents like ertapenem, cefepime, and teicoplanin, studies have shown consistent AUC/MIC and C_min_ values with SC administration, meeting therapeutic targets with minimal toxicity [6]. Still, regulatory and guideline frameworks lag behind clinical practice, and SC use remains off-label in many settings. This gap reinforces the need for pragmatic, evidence-informed approaches. Our narrative review aims to provide a practical and clinically grounded overview of SC antibiotic therapy. The literature review was conducted using several search strings, combining the terms “antibiotic”, “antimicrobial”, “subcutaneous administration”, “subcutaneous route”, and “subcutaneous injection” with appropriate Boolean operators.

## IDENTIFYING THE RIGHT PATIENT FOR SC ANTIBIOTIC THERAPY

IV, oral, and intramuscular (IM) routes are the most common administration methods in clinical practice. In hospital settings, IV therapy is predominant; however, it may be challenging in some patients [7]. While the IM administration can be alternative, its use is often limited by widespread anticoagulant therapy, poor tolerability for prolonged treatments, and delayed and/or unpredictable absorption [7, 8]. SC administration is easy to perform, reduces the need for venous access, and improves patient comfort, particularly in outpatient and palliative care settings [7, 9]. Where oral administration is not possible, establishing and maintaining IV lines can be challenging and carries risks of infection, thrombophlebitis, or mechanical complications. SC administration avoids these issues and is generally well-tolerated, with minimal training required for caregivers and patients alike [10]. Additionally, SC therapy may support outpatient antimicrobial therapy (OPAT) programs, allowing stable patients to receive treatment at home or in ambulatory settings [2, 11, 12]. Table 1 summarizes advantages and limitations of IM, IV, oral, and SC antibiotic therapy.

**Table 1.** Comparison Between IM, IV, Oral, and SC Antibiotic Therapy

Route	Advantages	Limitations
IM	Simple to deliver in resource-limited settings Avoids venous access	Painful Variable and sometimes delayed absorption;Limited volume per injection Risk of hematoma (esp. anticoagulation/thrombocytopenia) Not suitable for prolonged/high-frequency dosing
IV	Rapid onset Titratable exposureSuitable for severe/unstable infections Broadest evidence base	Requires venous access and monitoring Catheter-related infection/thrombosis risk Logistics/costs for OPAT
Oral	Convenient Cost-effective Noninvasive Facilitates early discharge	Bioavailability/drug–food–drug interaction issues; GI intolerance/malabsorption Adherence dependency; Not feasible with vomiting/ileus
SC	Patient-centred Minimally invasive Avoids venous access Feasible in OPAT and palliative care Stable exposure for time-dependent agents	Slower onset Local site reactions Absorption affected by peripheral perfusion/edema Off-label use Requires appropriate dilution/devices and staff training

GI, gastrointestinal; IM, intramuscular; IV, intravenous; OPAT, outpatient parenteral antimicrobial therapy; SC, subcutaneous.

## PHARMACEUTICAL CONSIDERATIONS FOR OPTIMIZING SC FORMULATION

Physical and biological aspects of the SC formulation play a key role in determining the efficacy of antibiotics administered via this route. These features must be considered alongside physicochemical and biological factors affecting SC absorption [13].

The main physical aspects include syringe ability, the ease with which a solution or suspension passes through a hypodermic needle, influenced by viscosity or density; resuspendability, the amount of shaking needed to resuspend settled particles; and good drainage, defined as clean separation of the suspension from the container walls [13].

Biological aspects concern in vivo performance, acknowledging that several variables influence PD response to an SC agent [13]. Key factors include particle size, solubilizers, viscosity, base/salt forms, and solvent effects [13]. Preclinical studies should also assess carcinogenesis, injection pain, and unexpected reactions to fully characterize biological response [13].

## PK/PD CHARACTERISTICS OF SC ANTIBIOTIC INFUSION

The “antimicrobial puzzle” concepts, optimizing antimicrobial dosing according to pathogen- and patient-specific factors such as bacterial susceptibility, infection site, patient pathophysiology, and drug-drug interactions, should also guide SC antibiotic administration [14, 15]. Achieving appropriate PK/PD targets for each agent remains essential [15]. From a PK/PD perspective, antibiotics may be roughly classified into (1) time-dependent agents (ie, beta-lactams), whose efficacy relies on %*f*T _>_  _MIC_; (2) concentration-dependent agents (ie, aminoglycosides), where efficacy depends on the C_max_-to-MIC ratio; (3) concentration-dependent agents with time dependence and postantibiotic effect (ie, glycopeptides, fluoroquinolones, oxazolidinones, daptomycin), for which efficacy correlates with *f*AUC/MIC [15].

**Figure 1.**
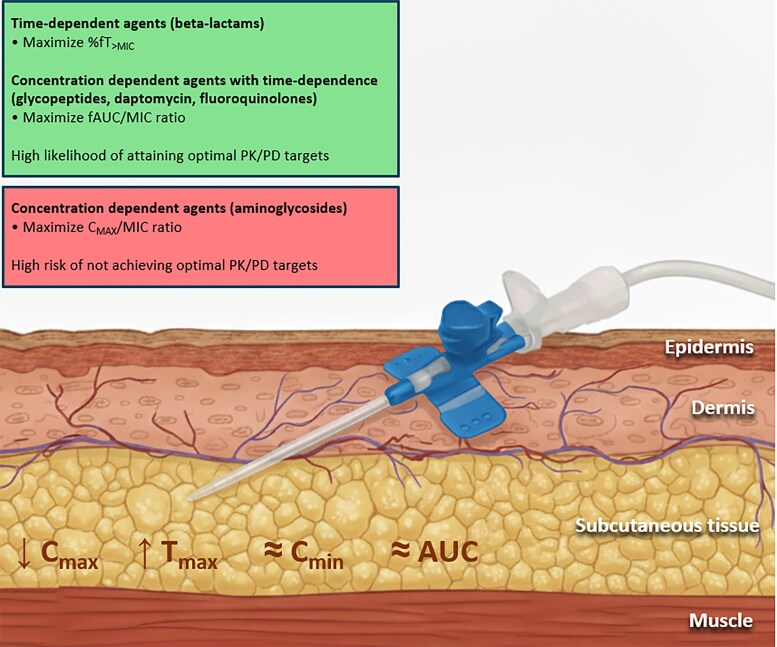
Relationship between general PK considerations with SC administration and PK/PD properties of different antibiotics.

Several studies report decreased C_max_ and delayed T_max_ with SC versus IV administration, while C_min_ and AUC_0–24_ remain similar (Figure 1, Table 2) [5, 9, 37, 38]. Consequently, SC administration is a valuable strategy for time-dependent antibiotics, whereas the risk of failing to meet PK/PD targets may be higher for concentration-dependent agents [5, 9].

**Table 2.** Main Studies Reporting PK Parameters of SC Antibiotics

Agent	n	Population	Study Design	Indication	Dose/Dilution SC	Dose/Dilution IV	SD/MD	AUC SC (mg·h/L)	Cmax SC (mg/L)	Cmin SC (mg/L)	Tmax SC (h)	t/2 SC (h)	Vd SC (L/kg)	CL SC (L/h)	AUC IV (μg·h/mL)	Cmax IV (mg/L)	Cmin IV (mg/L)	Tmax IV (h)	t/2 IV (h)	Bioav. (AUCSC/ AUCIV)	Vd IV (L/kg)	CL IV (L/h)	Ref
Ampi	22	HVs	Randomized crossover	PK ass.	1 g/50 mL NS in 20'	1 g/50 mL NS in 30'	SD (1-wk washout)	4527 ±1658	28 ±7	NA	0.75 ±0.3	1.4 ±0.4	29.7 ±14.3	4.33 ±2.28	3810 ±1033	49 ±11	NA	0.38 ±0.1	0.97 ±0.18	1.19	23.6 ±7.7	4.7 ±1.3	[16]
CZ	15	Noncritical HPs	Prospective crossover self-controlled study + PopPK	MSSA infection	1–2 g q6–8–12 h in 50 mL NS in 30’	1–2 g q6–8–12 h as per clinical indication	SD	NA	NA	NA	NA	NA	NA	NA	NA	NA	NA	NA	NA	74.2% (66.7–81.7%)	NA	NA	[4]
FEP	10	HVs	PopPK	PK ass.	1 g in 50 mL D5W in 30’	NA	SD	125.3	30.9	NA	NA	2.56	22.8	7.98	NA	NA	NA	NA	NA	NA	NA	NA	[17]
FEP	24 (12 SC vs 12 IV)	HPs	Multicenter retrospective case-control	Clinical indications	3.1 ± 1.8 g/day 1–2 g in 50 mL NS in 10–30’	3.3 ±1.9 g/d	MDs	NA	NA	29.05 (14.2–48.2)	NA	NA	NA	NA	NA	NA	31.9 (26.5–51.7) *P* > 0.05	NA	NA	NA	NA	NA	[18]
CAZ	89	HVs and pts with disabilities	PK study	PK ass.	0.5 g/10 mL in 30’ in two different sites	NA	SD	NA	44.8–57.4	NA	2.0–3.4	NA	NA	NA	NA	NA	NA	NA	NA	NA	NA	NA	[19]
CAZ	1	HP	Case report	Targeted therapy in PA HAP	2 g q8 h in 50 mL NS in 30’	NA	MDs	NA	NA	NA	NA	NA	NA	NA	NA	NA	NA	NA	NA	NA	NA	NA	[20]
CAZ	1	OP	Prospective	Suppr. in BJI	2 g in 50 mL NS in 30–45’	NA	MDs	NA	NA	5.6 ±0.4	NA	NA	NA	NA	NA	NA	NA	NA	NA	NA	NA	NA	[11]
CAZ	1	HP	Case report	Therapy in PA cUTI	1 g in 3 mL of 1% lidocaine solution	NA	MDs	NA	NA	31.1	NA	NA	NA	NA	NA	NA	NA	NA	NA	NA	NA	NA	[21]
CAZ	4	OPs	Case series	Suppr. in BJI	1–2 g in 50 mL NS in 30–45’	NA	MDs	NA	NA	2.0–22.4	NA	NA	NA	NA	NA	NA	NA	NA	NA	NA	NA	NA	[22]
CRO	10	HVs	Randomized crossover trial	PK ass.	0.5 g/2 mL 1% lidocaine solution	0.5 g/5 mL NS in 3’	SD	515 ±106	37.1 ±5.6	NA	NA	8.57 ±1.73	8.3 ±3.7	15.9 ±4.0	549 ±125	83.8 ±40.1	NA	NA	9.87 ±2.22	0.964 ±0.264	11.5 ±1.9	16.0 ±4.3	[23]
CRO	148 (110 iv vs 38 sc)	Elderly HPs	Retrospective	Therapy	1–2 g/d	1–2 g/d	MDs	NA	NA	NA	NA	NA	NA	NA	NA	NA	NA	NA	NA	NA	NA	NA	[24]
CRO	54	HVs	Phase I PK crossover study	PK ass.	1 g via rHuPH20-facilitated versus NS placebo	1 g in 30’	SD	1162.6 ±210.36 (rHuPH20) 1141.2 ±192.86 (placebo)	92.0 ±15.1 82.2 ±13.5	NA	2.02 (1.02–3.01) 3.02 (1.52–4.04)	7.97 (6.61–9.93) 8.28 (5.95–10.9)	NA	NA	1085.8 ±187.50	150.0 ±19.9	NA	0.502 (0.501–0.611)	8.25 (6.03–10.4)	106% (103–110%) 105% (101–108%)	NA	NA	[25]
CRO	3	OPs	Prospective	Suppr. in BJI	1 g in 50 mL NS in 30–45’	NA	MDs	NA	NA	NA	NA	NA	NA	NA	NA	NA	NA	NA	NA	NA	NA	NA	[11]
CRO	2	OPs	Case series	Suppr. in BJI	2 g in 50 mL NS in 30–45’	NA	MDs	NA	NA	6.6–71.7	NA	NA	NA	NA	NA	NA	NA	NA	NA	NA	NA	NA	[22]
CRO	47 (24 SC vs 23 IV)	HPs	PopPK	Therapy	1 g/d	1 g/d	MDs	1608 ±538 *P* > 0.05 versus IV	64.7 ±24.3 *P* < 0.001 versus IV	44.1 ±20.7 *P* > 0.05 versus IV	NA	NA	NA	NA	1621 ±409	146.3 ±37.8	39.8 ±16.2	NA	NA	99%	NA	NA	[26]
DAP	12	HVs	Randomized, crossover	Study	10 mg/kg in 50 mL NS in 30’	10 mg/kg in 50 mL NS in 30’	SD	937.3 ±102.5 (AUC0–24)	57.3 ± 8.6	19.8 ± 4.9	4 (3.5–4)	12 ± 3.1	NA	NA	1056.3 ± 123.5 (AUC0–24)	132.2 ±16.0	15.1 ± 3.5	0.5 (0.5–0.5)	9.3 ±1.7	0.87 ±0.10	5.43 (3.8% RSE)	0.62 (3.87% RSE)	[27]
ETP	7	OPs	Prospective	Suppr. in BJI	1 g in 50 mL NS in 30–45’	NA	MDs	NA	NA	2.88–28.3	NA	NA	NA	NA	NA	NA	NA	NA	NA	NA	NA	NA	[11]
ETP	26 (16 SC vs 10 IV)	HPs	Prospective + PopPK	Therapy	1 g/d in 30’	1 g/day in 30’	MDs	976.45	53 (15–145)	9 (3–27)	NA	NA	NA	1.02	1081	103 (28–149)	12 (2–20)	NA	NA	0.90	NA	0.95	[3]
ETP	4	OPs	Case series	Suppr. in BJI	1 g in 50 mL in NS in 30–45’	NA	MDs	NA	NA	1.0–43.9	NA	NA	NA	NA	NA	NA	NA	NA	NA	NA	NA	NA	[22]
FOS	1	Cystic fibrosis pediatric patient	Case report	Therapy: MDR PA and SA pneumonia	7200 mg/day (200 mg/kg/day) as a CI at concentration 300 mg/mL (1 mL/h)	NA	CI SC for 5 d (stopped for erythema and pain)	NA	NA	NA	NA	NA	NA	NA	NA	NA	NA	NA	NA	NA	NA	NA	[28]
MEM	11	Noncritical HPs	Prospective crossover self-controlled study + PopPK	Empirical or targeted therapy	1 g q8–12 h/50 mL NS over 30’	1 g q8–12 h/50 mL NS over 30’	SD	NA	< than IV in simulation	> than IV in simulation	NA	NA	21.7 (RSE 33%)	29.1 (RSE 18%)	NA	NA	NA	NA	NA	81% (71–93%)	NA	NA	[1]
MTZ	2	Patients scheduled for liposuction	Crossover	Surgical Prophylaxis	500 mg in 1.21L tumescent solution^a^, with 500 mg cefazolin (Pt 1) or 600 mg in 3.5L tumescent solution^a^, with 1200 mg cefazolin (Pt 2)	500 mg with 500 mg cefazolin (Pt 1) or 600 mg with 1200 mg cefazolin (Pt 2)	SD	81 (AUC0-∞, Pt 1) 81 (AUC0-∞, Pt 2)	4.8 (Pt 1) 4.8 (Pt 2)	NA	NA	NA	NA	NA	121.9 (AUC0-∞, Pt 1) 127 (AUC0-∞, Pt 2)	15 (Pt 1) 14 (Pt 2)	NA	NA	NA	NA	NA	NA	[29]
PEN	15	HVs	Randomized, crossover, PopPK	PK assessment	1.2MU over 2–3’	1.2MU over 2–3” (IM formulation)	SD (10-wk washout)	NA	36.3	10.5	NA	482.4	NA	NA	NA	56.8	5.2	NA	244.8	0.961 (0.893–1.06)	NA	NA	[30]
PEN	24	HVs	Phase I PK study	PK assessment	3.6MU/9mL 7.2MU/13.8mL 10.8MU/20.7mL	NA	SD	NA	NA	NA	NA	283.2 (259.2–307.2)	42.8 (40.0–46.1)	NA	NA	NA	NA	NA	NA	NA	NA	NA	[31]
TZP	448 (112 SC vs 336 IV)	HP	Retrospective case-control + propensity score	Empirical/targeted therapy	NA	NA	MDs	NA	NA	NA	NA	NA	NA	NA	NA	NA	NA	NA	NA	NA	NA	NA	[32]
TZP	1	HP	Case report	HAP—empirical therapy	4.5 g/50 mL NS	NA	MDs q6 h CI	NA	NA	Css d4: 52 Css d8: 93 Css d10: 156	NA	NA	NA	NA	NA	NA	NA	NA	NA	NA	NA	NA	[7]
TEC	12	Surgical ICU	Randomized crossover	Therapy	6 mg/kg maint. dose at day 4 after IV LD	6 mg/kg maint. dose at day 4 after IV LD of 6 mg/kg q12 h for 48h	SD	309 (180–640)	16 (9–31) *P* value <0.05 versus IV	10 (6–24)	7.5 (5–18) *P* < 0.05 versus IV	NA	NA	NA	369 (171–955)	70 (53–106)	9 (5–30)	0.5 (0.5–0.5)	NA	0.84	NA	NA	[33]
TEC	98	Gram-positive infections	PopPK	Therapy	6 mg/kg maint. dose at day 4 after IV LD	6 mg/kg maint. dose at day 4 after IV LD of 6 mg/kg q12 h for 48h	SD	352–1008 with simulated doses of 400–1000 mg/day (day 3)	NA	12–34 with simulated doses of 400–1000 mg/day (day 3)	NA	NA	97.4	0.305	450–1289 with simulated doses of 400–1000 mg/day (day 3)	NA	15–43 with simulated doses of 400–1000 mg/day (day 3)	NA	NA	97 ±14%	NA	NA	[34]
TMC	8	HVs	Randomized, Crossover	Study	2 g in 4.3 mL WFI and 1% lidocaine in 20’	2 g in 110 mL WFI in 40’	SD	818.1 ±90.3 (AUC0–12) 1314 ±242 (AUC0-∞)	100.0 ± 15.5	NA	4.8 ± 2.0	6.6 ± 1.9	0.18 ± 0.03	1.6 ± 0.3	959.4 ± 185.0 (AUC0–12) 1197.1 ± 276.6 (AUC0-∞.)	233.5 ±50.2	NA	0.67 ± 0.0	5.3 ±1.3	1.12 ±0.19	0.16 ±0.04	1.7 ±0.3	[35]
TMC	1	OP	Case report	cUTI prophylaxis	1 g/100 mL NS	NA	Suppr. therapy	NA	NA	9.5–16	NA	NA	NA	NA	NA	NA	NA	NA	NA	NA	NA	NA	[36]

Data are expressed as mean ± standard deviation or as median (interquartile range).

^a^(lidocaine [≤1 g/L], epinephrine [≤1 mg/L], sodium bicarbonate [10 mEq/L] in NS).

ass., assessment; AUC, area under the curve; Bioav., bioavailability; BJI, bone and joint infection; CAZ, ceftazidime; CZ, cefazolin; CI, continuous infusion; CL, clearance; C_max_, maximum plasma concentration; C_min_, minimum plasma concentration; CRO, ceftriaxone; C_ss_, steady-state concentration; cUTI, complicated urinary tract infection; D5W, dextrose 5% in water; DAP, daptomycin; ETP, ertapenem; FEP, cefepime; FOS, fosfomycin; HAP, hospital acquired pneumonia; HV, healthy volunteer; HP, hospitalized patient; ICU, intensive care unit; IM, intramuscular; IV, intravenous; LD, loading dose; MD, multiple dose; MDR, multidrug resistant; MEM, meropenem; MTZ, metronidazole; MU, million units; NA, not available; NS, normal saline; OP, outpatient; PA, *Pseudomonas aeruginosa*; PEN, penicillin G; PK, pharmacokinetic; Pt, patient; rHuPH20, recombinant human hyaluronidase; RSE, relative standerd error; SA, *Staphylococcus aureus*; SC, subcutaneous; SD, single dose; Suppr., suppressive therapy; t/2, half-life; TEC, teicoplanin; T_max_, time to reach maximum plasma concentration; TMC, temocillin; TZP, piperacillin/tazobactam; Vd, volume of distribution; WFI, water for injections; Wk, week.

A therapeutic drug monitoring (TDM)-guided approach may help ensure optimal PK/PD target attainment during SC antibiotic administration [5].

## SC ANTIBIOTIC ADMINISTRATION: TECHNICAL NOTES AND EXPERIENCES

Several anatomical sites are suitable for SC administration, including the abdominal wall, thigh, flank, peri-umbilical, peri-clavicular, peri-scapular, deltoid, hips, and occasionally the forearm or chest. Site selection should be individualized, as SC adipose thickness varies with age, sex, body composition, and anatomical region [39]. The chosen area must have intact skin and avoid bony prominences, joints, scars, intercostal spaces, edematous tissues, or poorly perfused regions [40]. Patient comfort, mobility, and adequate tissue (1–2.5 cm skinfold) should guide selection, to prevent intranuscular delivery [41 ]. The needle is inserted at a 45–90° angle depending on tissue depth [42, 43]. Metal needles are commonly used but should be discouraged because of the risk of local injury; plastic winged cannulas (eg, Teflon or polyurethane) are preferred [44–46]. Cannula gauge and length should match tissue thickness and drug formulation [42].

After insertion, aspiration must be performed to exclude blood reflux. If absent, the cannula should be flushed with ≥0.2 mL saline before administration [41, 47]. A transparent semipermeable dressing is advised to secure the device and allow inspection [48]. Sites should be monitored regularly and rotated based on infused volume, local tolerance, and patient comfort, though no standardized interval exists [49].

**Figure 2.**
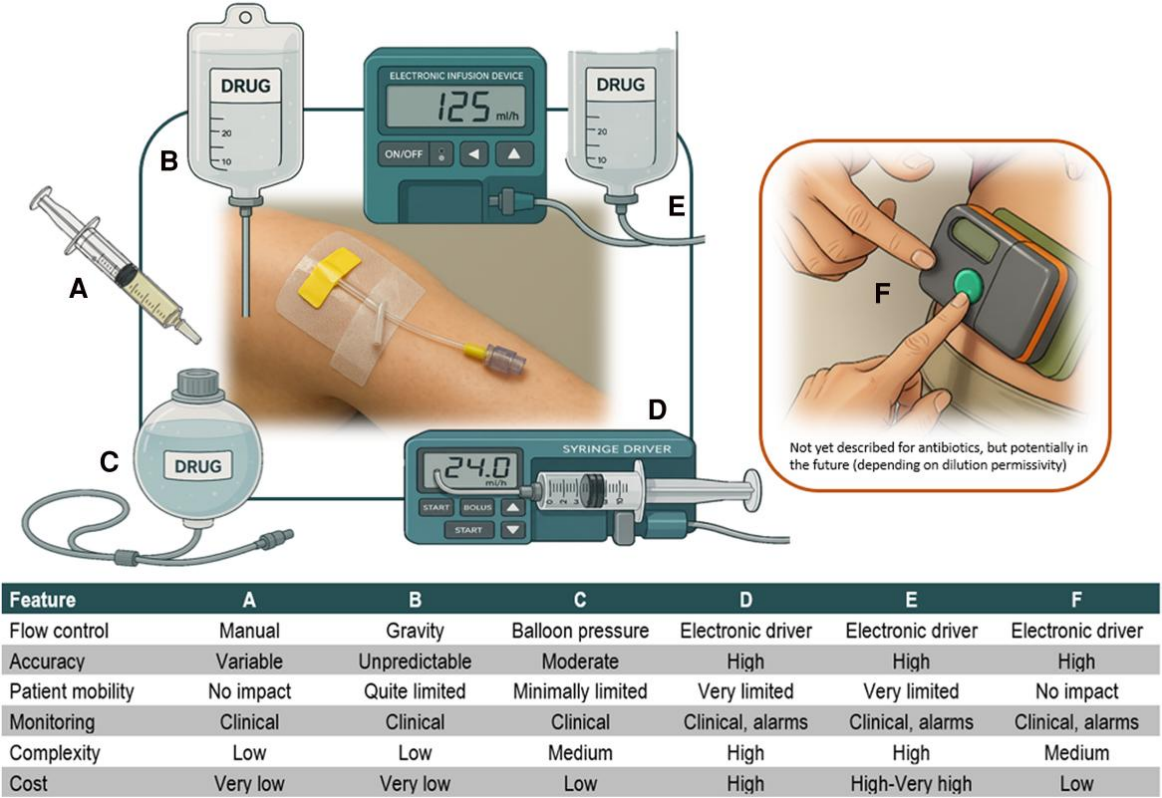
Comparison of different modalities for SC antibiotic infusion. (*A*) syringe bolus; (*B*) by gravity; (*C*) elastomeric pump; (*D*) syringe driver; (*E*) electronic infusion device; (*F*) on-body drug delivery system. The infusion rates shown are merely illustrative.

Drug delivery can occur via single injections or continuous infusions. Gravity, elastomeric devices, syringe or volumetric pumps may deliver infusions. For patient self-administration, wearable on-body SC delivery systems (eg, Enable Injections, Sonceboz, Sorrel Medical, West Pharmaceuticals, Ypsomed) are available; these are skin-adhered pumps typically placed on the arm, abdomen, or thigh (Figure 2).

Antibiotics are generally diluted in saline or glucose 5%, with final volumes ranging from 2 mL (bolus) to 100 mL (infusions). Bolus injections are given over 1–3 min, sometimes with lidocaine (0.5–2%, 1–5 mL) to reduce pain. Infusions (30–60 min) and are typically delivered through SC catheters (BD Saf-T-Intima, 22–25 G) or butterfly needles.

When exploring new SC antibiotic candidates, pH, osmolality, and formulation components must be evaluated to ensure tissue compatibility and stability. Nonphysiologic pH or osmolaritycan cause irritation, and excipients must be screened for local toxicity before clinical testing [50–52]. Several interventions may enhance tolerance and absorption, including lidocaine, hyaluronidase to improve diffusion, warm compresses before infusion, massage after injection to enhance perfusion, and postinfusion saline flushes.

## ANTIBIOTICS WITH SUPPORTING LITERATURE

### Penicillins

Despite their widespread use and favorable pharmacodynamics (%*f*T _>_  _MIC_), clinical data on SC penicillins remain limited. Penicillin G, in its benzathine form (BPG), SC administration has been explored only in syphilis and rheumatic heart disease prophylaxis. Two Phase I studies showed that SC BPG is safe, better tolerated, and achieves plasma levels comparable to IM administration [30, 31]. A 2023 study found that 10.8 MIU every 13 weeks provided PK similar to 1.2 MIU every 4 weeks [31].

SC ampicillin has been evaluated in a single study in elderly and young healthy volunteers, showing similar plasma levels to IV administration, albeit with delayed absorption [16].

SC piperacillin/tazobactam, despite its frequent inpatient use, remains poorly studied. In a 2019 retrospective analysis, 113 SC-treated patients were matched to 9113 IV-treated ones, with similar adverse event rates but higher all-cause mortality in the SC group [32]. A recent case report described successful treatment of hospital-acquired pneumonia with 10 days of SC piperacillin/tazobactam (4.5 g q6 h), with good tolerability and clinical resolution [7].

### Temocillin

Temocillin is an old narrow-spectrum β-lactam antibiotic increasingly used as an extended-spectrum beta-lactamases (ESBL)- and AmpC-resistant, carbapenem-sparing option for treating multidrug-resistant Enterobacterales infections [53]. In a randomized crossover trial in healthy volunteers, a single SC administration of 2 g temocillin produced a moderately lower AUC_0–12_ than IV, reflecting slower absorption and a flatter concentration-time profile typical of the SC route [35]. Notably, AUC_0-∞_ was 9.8% higher after SC injection, indicating a longer terminal exposure phase. Plasma levels remained above the PK/PD breakpoint throughout the 12 h interval, ensuring target attainment despite the blunted peak. These finding suggest that SC administration may be noninferior or even advantageous for maintaining sustained therapeutic levels over time. Under steady-state conditions, the sustained drug exposure from SC administration could result in more stable plasma concentrations and potentially reduced dosing frequency, supporting its use in long-term outpatient therapy. SC temocillin displayed acceptable tolerability, causing mild, transient local discomfort.

A case report described successful use of SC temocillin for prophylaxis of recurrent ESBL Enterobacterales urinary tract infections (UTIs) in a patient with difficult venous access [36]. Administered at 1 g once daily, this regimen maintained residual concentrations similar to those expected for curative use. More than 1 year of daily SC therapy, resulted in markedly fewer recurrences without local or systemic adverse reactions.

### Cephalosporins

Unlike penicillins, SC cephalosporins have been more extensively studied. SC ceftriaxone is the most investigated, particularly in France, with consistent PK and safety data [11, 23–25, 54]. While IV administration yields higher C_max_, other PK parameters are otherwise comparable [25]. A retrospective monocentric study found SC ceftriaxone to be as effective as IV in patients over 75 years (excluding septic shock), with better tolerability [24]. SC ceftriaxone has also been successfully used in prosthetic joint infections [11, 22]. Dosages varied (1–2 g/day), often following IV loading doses [55].

Clinical experience with other cephalosporins is more limited. SC cefepime appears safe, with PK values comparable to IM cefepime (1 g in healthy volunteers [17]), and similar clinical effectiveness to IV in a small retrospective study (average dose of daily SC cefepime: 3.1 g ± 1.8) [18]. SC ceftazidime was also well tolerated, with doses ranging 2–8 g/day, though efficacy data derive mainly from case reports and small series [11, 19–22].

SC cefazolin (1 or 2 g every 6 to 12 hours) was recently evaluated in 15 stable inpatients [4]. PK data and simulations using nonlinear mixed-effects modeling demonstrated lower peak and higher trough levels compared with IV cefazolin. Notably, a simulated dose of 6 g continuous 24 hours SC infusion achieved higher probability of target attainment (PTA) than 2 g every 8 hours at all MICs except 4 mg/L, suggesting continuous SC infusion might be feasible, and possibly preferred, for selected OPAT cases [4].

### Carbapenems

Carbapenems are typically reserved for severe infections, particularly in cases involving multidrug-resistant organisms, such as Gram-negative bacteria producing ESBL or AmpC [56]. Recent evidence has explored the feasibility of SC administration in selected scenarios.

Ertapenem is the most extensively studied for SC use due to its favorable PK profile, including a long half-life and low infusion volume. Clinical data indicate that SC ertapenem achieves comparable serum concentrations to IV, with good local tolerability and minimal adverse effects [9–11]. Doses ranging from 500 mg daily to 1 g twice daily, have been used for joint, respiratory, urinary and surgical site infections, with treatment success rates between 57% (chronic bone and joint infections [BJIs]) and 100% [3, 10, 11].

SC meropenem has also been reported in small studies. Murray *et al* demonstrated that 1.5 g twice daily or 3 g as a 24 h SC infusion yielded lower C_max_ but higher C_min_ than IV administration. Free drug concentrations exceeded the MIC for > 40% of the dosing interval more frequently with SC than IV at MICs 0.03–8 mg/L [1].

Data on SC imipenem therapy in humans remain limited. Some animal models suggest favorable PK/PD properties, but clinical evidence is lacking [57].

### Glycopeptides

Teicoplanin is a glycopeptide active against staphylococcal and enterococci, with a long half-life (24–48 hours) that makes it suitable for outpatient management of Gram-positive infections [58]. Several studies support its use via the SC route [10, 33, 34, 59, 60]. A population PK study of 98 patients with documented Gram-positive infections showed a teicoplanin SC bioavailability of 97 ± 14% compared with IV [34]. The SC route was associated with lower initial C_min_ and AUC after loading dose compared with the IV route [34]. However, after 14 days, the simulated PTA for all tested dosages in terms of C_min_ and AUC/MIC ratio, was comparable between the two routes [34]. A randomized crossover study in 12 surgical ICU patients compared IV ans SC teicoplanin after an IV loading dose [33]. Median C_min_ (10 vs 9 mg/L), AUC_0–24_ (309 vs 369 mg×h/L), and the percentage of the dosing interval with concentrations >10 mL/L (96% vs 79%) were similar, confirming PK/PD equivalence [33]. In other studies, SC teicoplanin was not associated with an excess of failure in patients affected by staphylococcal BJIs (clinical cure rate 58–72%) [10, 59, 60]. Furthermore, no safety issues were reported with the SC administration of teicoplanin [59].

No PK or clinical data are currently available for SC administration of vancomycin.

### Fosfomycin

Fosfomycin exhibits broad-spectrum activity against Gram-positive and Gram-negative bacteria [61] and is often used, alone or in combination, for managing difficult-to-treat infections [62]. SC experience is limited, and evidence remains scarce. A case report described SC fosfomycin for a respiratory exacerbation in a pediatric cystic fibrosis patient. The clinical response was favorable, but local tolerance was suboptimal, with irritation, swelling, and pain at the injection site leading to two treatment interruptions. No severe adverse reactions were reported [28 ].

SC fosfomycin administration may represent a feasible alternative, but further investigation is required.

### Daptomycin

Daptomycin is a cyclic lipopeptide with concentration-dependent bactericidal activity, where AUC/MIC as the key PK/PD parameter. It is mainly used as a reserve agent against Gram-positive pathogens [63]. Its once-daily dosing makes SC administration potentially convenient for outpatient therapy. In a randomized crossover study in healthy volunteers receiving 4 mg/kg daptomycin, SC administration resulted in a moderately reduced AUC_0–24_, and delayed T_max_ compared with IV dosing [27]. Nevertheless, systemic exposure remained within the bioequivalence range for the AUC_0–24_ defined by EMA criteria [64], and the PTA met predefined PD targets for both administration routes. The incidence of adverse events was significantly higher in the daptomycin group compared with the SC placebo control (NaCl 0.9%), though the events were predominantly mild and localized. Importantly, daptomycin is commonly administered at higher doses (>8 mg/kg) in clinical practice. Therefore, additional studies are necessary to evaluate the safety of higher doses for subcutaneous administration [65].

### Dalbavancin

Dalbavancin is a lipoglycopeptide with potent activity against a broad range of Gram-positive pathogens. Its long half-life permits once-weekly dosing, making it especially appealing for outpatient or suppressive therapy [66, 67]. Evidence on SC use is limited to a single case report, in which an elderly patient with *Enterococcus faecium* prosthetic joint infection received two monthly SC injections [68]. Therapeutic plasma concentrations were achieved, and treatment was well tolerated apart from mild local swelling. These observations suggest that dalbavancin's PK properties may support SC administration and warrant further clinical investigation.

### Other Antibiotics

For several antibiotic classes, data on SC administration are limited or indicate poor local tolerability. Several agents offer no clinical advantage via the SC routebecause effective oral formulations already exist or their PK/PD properties are poorly suited to SC delivery. In particular, antibiotics with concentration-dependent activity (eg fluoroquinolones and aminoglycosides), are unlikely to meet optimal PK/PD parameters given the attenuated peak levels following SC injection [69]. Local tolerability is another concern: studies have reported injection site reactions with fluoroquinolones and skin necrosis with SC aminoglycoside administration [70–74]. No human data exist on the SC administration of macrolides or linezolid. However, both macrolides and linezolid also have effective oral formulations with high bioavailability. Similarly, tetracyclines and rifamycins lack clinical data on SC use. Though direct evidence on local toxicity is sparse, both classes are known to cause irritation and tissue injury when administered parenterally, and extravasation should be avoided [75–77]. There are no published data on the SC administration of polymyxin B or colistin in humans; however, the lack of an oral formulation and the feasibility of once-daily dosing make the SC route an attractive option worth investigating (with attention to local tolerability) [78]. Metronidazole is widely used for surgical prophylaxis [79]. One French study reported SC administration in 16 patients, though no further data on efficacy or safety were provided [6]. Additionally, a study using Tumescent anesthesia antibiotic delivery, a technique involving SC infiltration of antibiotics in tumescent lidocaine, demonstrated acceptable local tolerability of metronidazole [29].

Importantly, the tolerability of SC administration is not a class effect. Even when multiple antibiotics within a class show promising results for SC delivery new agents require cautious evaluation in preclinical and early clinical trials to ensure safety and tolerability. The evidence landscape and practical guidance for SC antibiotic administration based on current literature are summarized in Table 3.

**Table 3.** Evidence Landscape of SC Antibiotic Administration

Antibiotic	Common Administration Mode	Typical Dosage^a^	Main Potential Indications	Notes
Ceftriaxone ■■■	1–2 g in 50 mL NS in 30’^b^	q12–24h	BJI; other indications as per IV ceftriaxone	AUC and C_min_ comparable to IV; robust PK studies; less adverse events than IV
Ertapenem ■■■	1 g in 50 mL NS in 30’	q24h	BJI, cUTI, ESBL infections	Good PK/PD, acceptable local tolerability
Teicoplanin ■■■	6–8 mg/kg in 50 mL NS in 30’	q24h	Staphylococcal/enterococcal BJIs	PK/PD similar to IV; robust studies; ulcer risk with >600 mg/day; SC after IV LD
Cefazolin ■■□	1–2 g in 50 mL NS in 30’	q8h	MSSA infections	Favorable bioavailability (74.8%) and target attainment; well tolerated; worth investigating as SC CI
Ceftazidime ■■□	2 g in 50 mL NS	q8h	BJI, *Pseudomonas* HAP and UTI	Generally well tolerated; time-dependent PK preserved
Daptomycin ■■□	10 mg/kg in 50 mL NS in 30’	q24h	MRSA/VRE infections	Common mild reactions; transient; delayed T_max_ but AUC comparable to IV
Meropenem ■■□	1 g in 50 mL NS	q8h	ESBL infections	Limited published experience; short half-life
Temocillin ■■□	2 g in 4.3 mL WFI + 1% lidocaine	q8–12h	ESBL infections, UTI	Case reports suggest feasibility
Ampicillin ■□□	1 g in 50 mL SF	q4h	Poorly suited drug for SC	High injection volume; generally inconvenient for SC use
Cefepime ■□□	1–2 g in 50 mL NS in 30’	q8h	*Pseudomonas*, AmpC infections	Similar PK to IM; limited experience
Dalbavancin ■□□	500 mg in 25 ml WFI + 75 mL D5W (divided into two 50 mL syringes in 45’)	≥weekly	Gram-positive infections	Only one case report (interesting molecule for SC use)
Fosfomycin ■□□	Concentration 300 mg/mL, given in CI^c^	CI	Pneumonia, UTI	Only one case report (local pain, erythema after some days)
Metronidazole ■□□	500 mg in 1.2 L tumescent solution	Single dose	Surgical prophylaxis	Investigated for surgical prophylaxis; acceptable safety
Penicillin G (benzathine) ■□□	3.6 MU/9 mL; 7.2 MU/13.8 mL/10.8 MU/20.7 mL (pre 2 mL 1% lignocaine)	Single dose	SSTI prophylaxis; syphilis (theoretical)	Well tolerated; simulations suggest 3 m therapeutic levels for syphilis
Piperacillin/tazobactam ■□□	4.5 g in 50 mL NS in 30’	q6–8h	Nosocomial infections	Few case reports; well tolerated; only mild local reactions
Aminoglycosides □□□	Limited human data; limited safety data (high risk of necrosis reported); PK/PD unfavorable for SC			
Fluoroquinolones □□□	Limited human data; limited safety data (necrosis reported); PK/PD unfavorable for SC			
Vancomycin □□□	Limited human data; high osmolarity and tissue irritation potential			
Colistin/Polymyxin B ○	No human data; attractive option to investigate (with attention to local tolerability)			
■■□ safe, highly experienced, included in guidelines	■■□ safe, highly experienced, included in guidelines	■□□ probably safe, but limited data on dosing and efficacy	□□□ best avoided except in extreme/exceptional cases	○ not yet studied (theoretically promising)

^a^Normal renal function; AUC: area under the curve.

^b^Better split into multiple sites if >1 g.

^c^In one pediatric patient.

BJI, bone and joint infection; CI, continuous infusion; D5W, dextrose 5% in water; ESBL, extended-spectrum beta-lactamases; HAP, hospital acquired pneumonia; IM, intramuscular; IV, intravenous; LD, loading dose; MRSA, methicillin-resistant Staphylococcus aureus; NS, normal saline; PK/PD, pharmacokinetic/pharmacodynamic; SC, subcutaneous; SSTI, skin and soft tissues infection; Tmax, time to reach maximum plasma concentration; UTI, urinary tract infection; VRE, vancomycin-resistant Enterococcus; WFI, water for injections.

## EXPECTED AND UNWANTED COMPLICATIONS

During SC administration, localized swelling at the injection site, proportional to volume and infusion rate, is expected and reflects the slower absorption profile. This typically resolves as the antibiotic diffuses into surrounding tissues.

Overall, complications are predominantly local and usually mild, with no reported cases of bacteremia or invasive infections attributable to SC delivery [5]. Documented reactions include warmth, erythema, pain, pruritus/urticaria, hypesthesia, bruising, burning, lymphangitis, and superficial fungal infection. These reactions were usually transient, occurred mainly after the first infusion, and rarely required discontinuation administration. While they occasionally prompted a change of site, they rarely interfered with daily activities, prolonged hospital stay, or required discontinuation of therapy.

Adverse events appear related not only to the antibiotic class but also to modifiable factors, such as hypotonic glucose or lidocaine diluents, drug concentration, infusion rate, and use of rigid steel needles [6, 28, 80, 81]. To reduce injection-related pain, co-administration of a local anesthetic (eg, lidocaine 1%) has been suggested, improving tolerability [23]. However, rare but severe adverse events such as skin necrosis have been reported, particularly with gentamicin [72–74, 82] and amikacin [73].

Notably, in comparative studies, the incidence of local or systemic adverse events following SC administration was similar to, or in some cases lower than, that observed with IV or IM routes.

For home-based treatment, patients and caregivers should be advised to inspect the site twice daily and promptly report any discharge, pain, erythema, bruising, burning, or persistent swelling to their healthcare provider.

## TOLERABILITY AND PATIENTS EXPERIENCE

Most tolerability data are observational, heterogeneous in populations and often focus on clinician-reported local events rather than validated patient-reported measures [6].

A recent prospective observational study specifically assessed the tolerance of SC ceftriaxone in older adults (≥75 years) using a structured patient-reported pain questionnaire [83]. Pain was evaluated through the Algoplus scale, validated for geriatric patients, before, during, and after infusion. In parallel, nurses also documented local adverse events through dedicated questionnaires. More than half of participants (57%) reported some degree of pain, mainly related to the introduction of the catheter.

More recently, the safety and tolerability of SC cefazolin [4] and meropenem [1] have been evaluated in two prospective, crossover, self-controlled studies. Pain was measured by means of a numerical rating scale (NRS) from 0 to 10 at predefined timepoints during and after the SC infusion. Local skin reactions were evaluated through visual inspection and graded on a 0–4 scale for erythema and edema. Across both studies, SC administration was well tolerated. Most participants reported no pain or only mild transient discomfort (NRS ≤3) during infusion, with no residual pain after completion. Mild, self-limiting local edema was occasionally observed but resolved completely within two hours, and no erythema or induration was reported.

## REGULATORY CONSIDERATIONS AND FUTURE PERSPECTIVES

To our knowledge, no scientific society has established guidelines regarding SC administration of antibiotics. Furthermore, SC administration is not registered by any national medical/medicinal agency. Despite this, antibiotics continue to be administered subcutaneously [84, 85]. Until 2014, SC administration of ceftriaxone had been registered by the French national medication agency (ANSM). However, the authorization was revoked after the EMA harmonized ceftriaxone package inserts across Europe. Since then, SC administration of ceftriaxone is classified as off-label also by the ANSM.

Regarding future perspectives: further technological innovations such as recombinant hyaluronidase (rHuPH20), wearable drug delivery systems and microneedle array (MNA) technology, may improve patient comfort and/or PK parameter attainment of antibiotics administered subcutaneously.

Since SC antibiotic delivery results in a slow diffusion from soft tissue to the bloodstream, the adjunction of rHuPH20 to SC antibiotics is thought to improve the PKs of SC antibiotics. This was confirmed in one study including 54 healthy young volunteers; SC ceftriaxone combined with a rHuPH20 compared with SC ceftriaxone with placebo and IV ceftriaxone achieved faster and higher C_max_ and less local swelling [86].

Wearable SC drug delivery systems already used to administer monoclonal antibodies or granulocyte colony-stimulating factor could be used for OPAT, offering increased patient autonomy and reduced need for catheter manipulation [87].

Beyond traditional SC injection via catheters, MNA technology, devices containing microscopic needles that cross the epidermis to release drugs in a controlled and painless manner, provides an innovative transdermal administration route. Although data on MNA administered antibiotics are limited to animal and ex-vivo data, potential applications include the treatment of wound infections and neonatal sepsis [26, 88].

## CONCLUSIONS

SC antibiotic administration is a practical alternative to the IV route in selected patients. Available data show that several time-dependent agents, including β-lactams and teicoplanin, achieve PD targets with acceptable safety. This option is particularly relevant for patients with poor venous access, in palliative care, and in outpatient settings, where it may reduce catheter-related complications and improve comfort. Conversely, concentration-dependent antibiotics provide less predictable exposure and should be used with caution. Clinical reports and PK studies confirm feasibility, but evidence remains heterogeneous. Except for French recommendations, no society guidelines currently formally address this practice, and regulatory frameworks are lacking. Further trials and real-world evaluations are required to define optimal agents, dosing strategies, and patient selection. In the context of antimicrobial stewardship and resource optimization, the SC route offers an additional tool to expand treatment possibilities while minimizing invasive procedures. Careful integration into clinical practice, supported by robust evidence, will determine its role in future infectious disease management.
